# MEMS-Based Sensor for Simultaneous Measurement of Pulse Wave and Respiration Rate

**DOI:** 10.3390/s19224942

**Published:** 2019-11-13

**Authors:** Thanh-Vinh Nguyen, Masaaki Ichiki

**Affiliations:** Sensing System Research Center, National Institute of Advanced Industrial Science and Technology (AIST), Ibaraki 305-8564, Japan; ichiki-m@aist.go.jp

**Keywords:** pulse wave, respiration, microelectromechanical systems (MEMS), piezoresistive, cantilever, tube

## Abstract

The continuous measurements of vital signs (body temperature, blood pressure, pulse wave, and respiration rate) are important in many applications across various fields, including healthcare and sports. To realize such measurements, wearable devices that cause minimal discomfort to the wearers are highly desired. Accordingly, a device that can measure multiple vital signs simultaneously using a single sensing element is important in order to reduce the number of devices attached to the wearer’s body, thereby reducing user discomfort. Thus, in this study, we propose a device with a microelectromechanical systems (MEMS)-based pressure sensor that can simultaneously measure the blood pulse wave and respiration rate using only one sensing element. In particular, in the proposed device, a thin silicone tube, whose inner pressure can be measured via a piezoresistive cantilever, is attached to the nose pad of a pair of eyeglasses. On wearing the eyeglasses, the tube of sensor device is in contact with the area above the angular artery and nasal cavity of the subject, and thus, both pulse wave and breath of the subject cause the tube’s inner pressure to change. We experimentally show that it is possible to extract information related to pulse wave and respiration as the low-frequency and high-frequency components of the sensor signal, respectively.

## 1. Introduction

Pulse wave and respiration rate are among the four fundamental vital signs in human beings, which indicate the state of a body’s life-sustaining functions. In recent years, there has been growing interest in the use of wearable devices for continuous measurement of pulse wave and respiration rate instead of the traditional manual measurements performed by healthcare professionals. In fact, the development of wearable devices that can measure pulse wave and respiration rate has been an important research topic, with practical applications in the fields of healthcare, security, and sports [1,2]. Furthermore, continuous pulse wave measurements can be used to develop methods for continuous monitoring of blood pressure [3,4,5,6].

State-of-the-art wearable devices for pulse wave measurement can be divided into two: Mechanical- and optical-based devices. Mechanical-based devices measure pulse waves by measuring the deformation of the skin above the blood vessel using force and pressure sensors [7,8,9,10,11], whereas optical-based devices use a light emitting diode (LED) to illuminate the skin and then use a photodiode to detect the amount of light transmitted or reflected from the skin’s surface to calculate pulse waves [12,13]. Furthermore, for measurement of respiration rate, various wearable devices that can be attached to an area close to the nose, neck, or chest of a subject were previously proposed. These sensors determine the respiration rate by detecting changes in nasal airflow, respiratory sound, exhaled air temperature, exhaled air humidity, and CO_2_ concentration in exhaled air [14,15,16]. However, for these approaches to successfully work, the wearable sensors have to be attached to a certain part of the body or to a mask, which would typically cause discomfort to users, especially when they are participating in outdoor sports or exercising.

Moreover, because wearing multiple devices will cause even more discomfort to users than wearing only one, a method that can simultaneously measure multiple vital signs using only one device, including pulse wave and respiration rate, is highly desirable. However, in general, most conventional methods for measuring multiple vital signs, such as pulse wave and respiration rate, often involve the use of two or more specific measurement devices. Thus far, only methods for simultaneous measurements of heart and respiration rates have been reported. One such approach involves extracting respiration rate information from the photoplethysmogram (PPG) measured via an optical-based method for pulse wave measurement, as described above [17,18]. However, because the PPG response could be altered by several factors, such as ambient light, muscle movement, and skin dilation [19], this method is not suitable for use during motion of the body, including physical exercise. Another approach involves the use of radar to detect the deformation of the chest due to both heartbeat and respiration [20,21]. However, this method is also not suitable for measurement during outdoor activities because it requires the measurements to be performed in a designated space with radar equipment.

**Figure 1:**
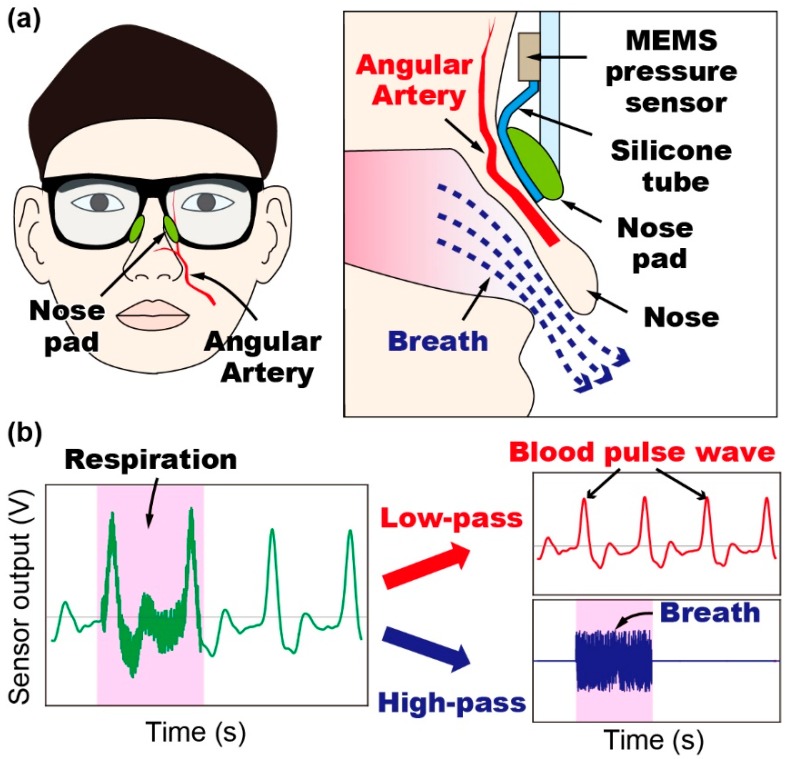
(**a**) Conceptual schematic diagram of the proposed method to simultaneously measure blood pulse wave and respiration rate using a single sensor device attached to the nose pad of eyeglasses. (**b**) Sensing principle: The pulse wave and respiration rate are measured using the low-pass filtered and high-pass filtered signals of the output of the proposed sensor, respectively. (MEMS: MicroElectroMechanical Systems).

In this study, we propose a method to simultaneously measure pulse wave and respiration rate using a tube-shaped microelectromechanical systems (MEMS)-based pressure sensor attached to the nose pad of a pair of eyeglasses, as shown in Figure 1a. The pulse wave and respiration rate of the subject wearing the eyeglasses are measured as the filtered signals derived from the sensor output passing through low-pass and high-pass filters, respectively (Figure 1b). We select eyeglasses as the device medium for sensor integration because they do not cause distraction to the wearer during the measurements. In this paper, we present the sensing principle and sensor design, as well as the fabrication and experimental details of our proposed prototype device for simultaneous measurement of pulse wave and respiration rate.

## 2. Materials and Methods

**Figure 2:**
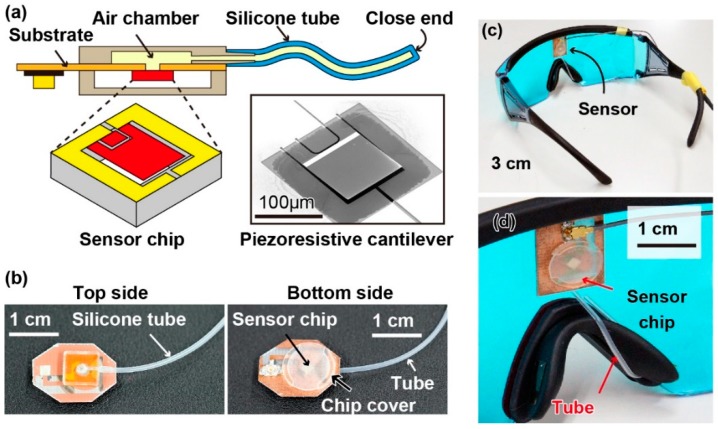
(**a**) Structure of the proposed sensor device with a silicone tube attached to an air chamber with a piezoresistive cantilever inside. (**b**) Photographs of the fabricated sensor device. (**c**,**d**) Photographs of the eyeglasses with the proposed sensor device. The tube is attached to the nose pad of the eyeglasses.

The proposed sensing principle is illustrated in Figure 1b. In our method, we used the vibration of the skin on the nose caused by breath and pulse wave in the angular artery to measure respiration rate and pulse. In particular, we used a tube-shaped differential pressure sensor attached to the nose pad of a pair of eyeglasses to measure this vibration. Because of the difference in the frequencies of the vibrations caused by the breath and pulse wave, these vital signs could be separately extracted from the sensor signal. The structure of the proposed device is shown in Figure 2a. A piezoresistive cantilever was used to measure the change in the tube’s inner pressure. The sensing principle of the cantilever was based on the piezoresistive effect: The resistance of the cantilever changes when it bends. The fabrication process for the cantilever used in our device is reported elsewhere [22,23,24,25,26]. A scanning electron microscope (SEM) image of the fabricated cantilever is shown in Figure 2a. The cantilever was 125 μm long, 100 μm wide, and 0.3 μm thick. To assemble the device, first, the sensor chip was attached to a thin substrate with patterned copper electrodes using glue. The sensor chip was then wire-bonded to the electrodes and a three-dimensional (3D)-printed cap was glued to the substrate to cover the sensor chip. Next, a 3D-printed chamber connected to a silicone tube was attached to the backside of the substrate. Because there was a through-hole underneath the sensor chip, the cantilever could measure the air pressure change that occurs inside the tube. The photographs of the fabricated device are shown in Figure 2b. Finally, the fabricated sensor device was attached to a pair of eyeglasses and the tube was wound into the nose pad of the eyeglasses, as shown in Figure 2c,d, respectively. It should be noted that in the current prototype device, electrical wires were used to connect the cantilever with the outer circuit. Furthermore, the tube was made of silicone rubber whose Young’s modulus was approximately 1 MPa. The inner dimeter, outer diameter, and length of the tube were 0.5 mm, 1 mm, and 4 cm, respectively. The tube was knotted to create a closed chamber underneath the cantilever, and the volume of the chamber could be adjusted by changing the position of the knot. Moreover, because the tube was soft and thin, it did not cause discomfort to users.

## 3. Results

### 3.1. Sensor Calibration

**Figure 3:**
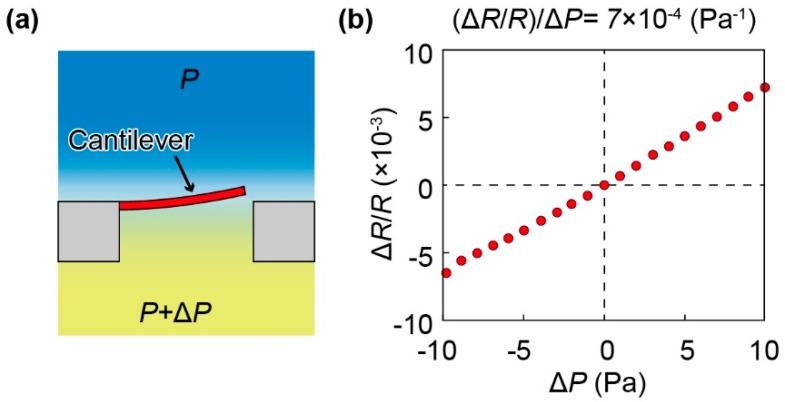
Calibration of the cantilever: (**a**) Conceptual diagram of the calibration method. (**b**) Relationship between the fractional resistance change of the sensor and applied differential pressure on the cantilever.

The cantilever was calibrated using the method illustrated in Figure 3a. In particular, a pressure calibrator (Kal-200, Halstrup-walcher GmbH, Germany) was used to apply and control the differential pressure on the top and bottom surfaces of the cantilever. The change in resistance of the cantilever was measured using a setup consisting of a Wheatstone bridge circuit and an amplifier. The output of the amplifier was recorded using a scope-coder (DL850, Yokokawa Inc., Tokyo, Japan). The calibration result is shown in Figure 3b. From this result, the relationship between the fractional resistance change of the cantilever Δ*R*/*R* and applied differential pressure Δ*P* was obtained as follows:

(1) $$\frac{∆R}{R}=7\times 10^{-4}∆P,$$

Because our measurement setup was able measure a fractional resistance change of 8 × 10^−6^, the sensing resolution of the cantilever was calculated to be approximately 0.01 Pa.

### 3.2. Measurement of Pulse Wave

**Figure 4:**
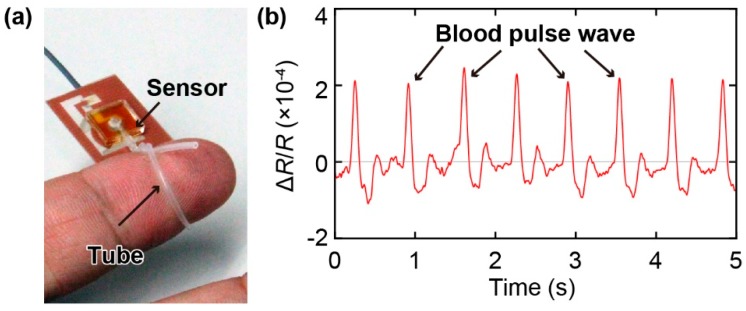
Demonstration of pulse wave measurement. (**a**) Photograph of the tube of the sensor wound around an index finger. (**b**) Response of the sensor to the pulse wave. The signal of the sensor was low-pass filtered with the cut-off frequency of 5 Hz.

Next, we demonstrate the ability of the sensor to measure pulse waves. Previously proposed wearable devices developed for pulse wave measurement had to be placed exactly on a blood vessel to detect the pulse wave. However, with the tube-shaped sensor proposed in this study, pulse wave measurement can be achieved by winding the tube around a finger (Figure 4a). The results shown in Figure 4b indicate that not only is the proposed sensor sufficiently sensitive to measure the pulse wave, but it can also be easily attached to the wearer without the necessity to carefully align it with a blood vessel. Thus, the proposed sensor is suitable for the development of a ring or bracelet-type wearable sensor for pulse wave measurements.

### 3.3. Simultaneous Measurements of Pulse Wave and Respiration

**Figure 5:**
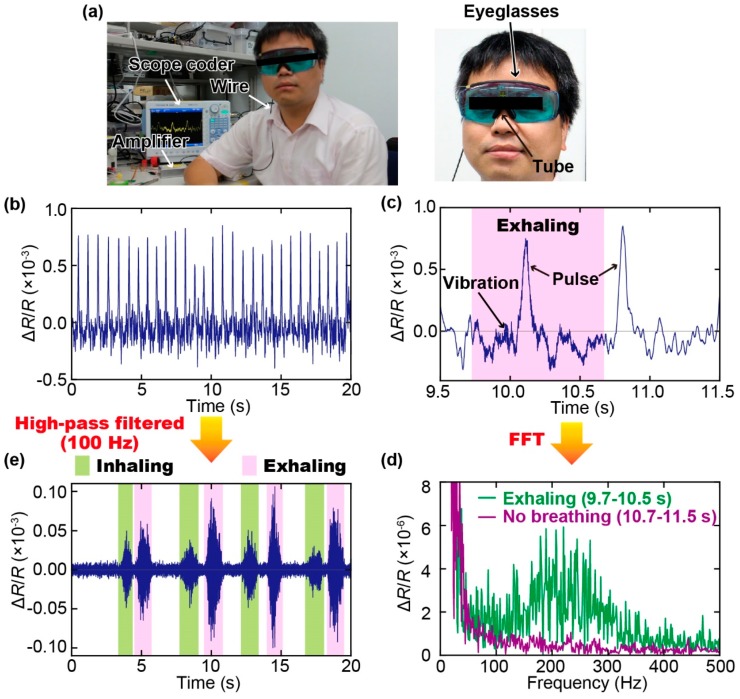
(**a**) Photograph of the experimental setup for simultaneous measurement of pulse wave and respiration rate using the fabricated smart eyeglasses. (**b**) Output of the sensor recorded for 20 s, which clearly shows the pulse waves. (**c**) Zoomed-in view of the sensor output showing the parts with and without respiration. (**d**) Frequency characteristics of the sensor signals with and without respiration. (**e**) Sensor signal after being filtered through a 100-Hz high-pass filter, which clearly shows the inhaling and exhaling processes by the subject.

Finally, we performed simultaneous measurement of the pulse wave and respiration using the proposed sensor attached to a pair of eyeglasses, as shown in Figure 2c. The experimental setup is shown in Figure 5a. A subject (healthy male, 34 years old) was asked to wear the eyeglasses and breathe normally for 20 s. During the measurement, the change in resistance of the cantilever was recorded at a sampling rate of 1000 samples.

**Figure 6:**
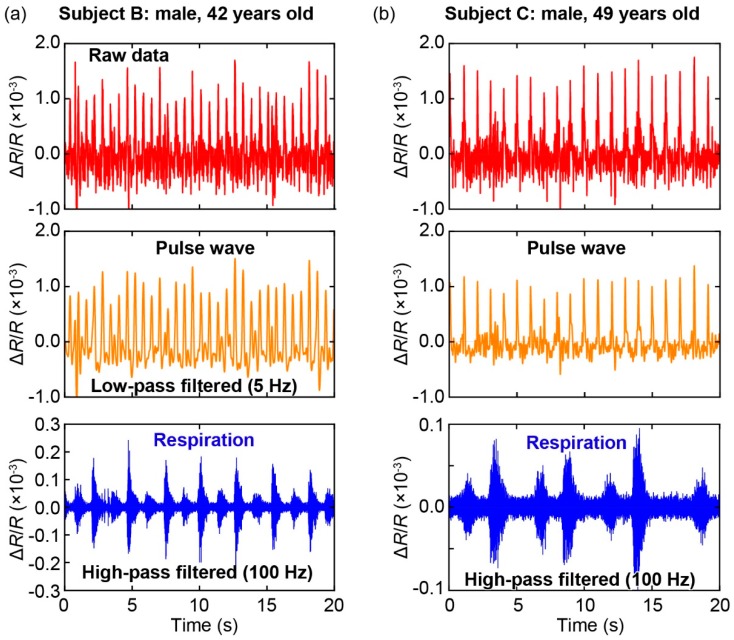
Simultaneous measurements of the pulse wave and respiration on two other subjects. The results are similar to those in Figure 5.

The change in resistance of the cantilever during our measurement is shown in Figure 5b. Furthermore, a zoomed-in view of the sensor output is shown in Figure 5c, which clearly indicates that the pulse wave could be measured using the proposed sensor. Moreover, the difference can be clearly observed in the regions with and without respiration activity: The part of the sensor output during breathing by the subject included more fluctuations compared to that with a pause, owing to the vibration induced by breathing. By using Fast Fourier Transformation (FFT), the frequency characteristics of the sensor outputs with and without respiration can be obtained, as shown in Figure 5d. Our results show that respiration-induced vibration has a frequency range of approximately 100–400 Hz. Therefore, the respiration rate of a subject can be extracted from the signal of the sensor using a high-pass filter with a cut-off frequency of 100 Hz. Figure 5e shows the filtered signal, wherein inhaling and exhaling by the subject were clearly observed. In addition, the results highlight that the exhaling motion generally induced a stronger vibration than the inhaling motion did. Moreover, Figure 6 shows the results of the measurements on two other subjects (healthy males, 42 years old (Figure 6a) and 49 years old (Figure 6b)) using the same sensor device. It is confirmed that the pulse waves and respirations of the subjects could be extracted from the signals of the sensors using a low-pass filter (cut-off frequency: 5 Hz) and a high-pass filter (cut-off frequency: 100 Hz), respectively.

## 4. Discussion

In this study, we proposed a method to simultaneously measure respiration rate and pulse wave using a single sensor device with a tube-type MEMS-based pressure sensor. The pressure sensor used in this study is a cantilever-type sensor, which can measure a differential pressure as small as 0.01 Pa. The cantilever structure makes it difficult to measure the static pressure change or pressure change at low frequency. However, by making the gap extremely small, it is possible to lower the frequency range of pressure change that can be detected by the sensor. In the proposed sensor device, the gap between the cantilever and surrounding wall is approximately 1 μm, which allows the cantilever to measure differential pressure at low frequency (0.1 Hz) [27]. These advantages make the cantilever quite suitable for measurement of vibrations in the body induced by vital signs. However, because the cantilever is small and fragile, it cannot be brought in direct contact with the skin to measure these vibrations. Therefore, in this study, we integrated the cantilever with an air cavity, which, in turn, was connected to a tube. This structure allowed for the measurement of vibrations applied to any location on the tube. Moreover, because the air could go through the gap around the cantilever and it was possible to stretch or wind the tube without breaking the cantilever. Therefore, the structure proposed in this study is suitable for development of wearable devices for monitoring pulse wave, blood pressure, and respiration rate. Furthermore, the tube shape makes it easy to attach the sensor to the nose pad of a pair of eyeglasses, and it would cause little distraction to the wearer. Thus, the proposed device is suitable for continuous health monitoring in various applications, such as for the well-being of workers in a factory, in sport training, and for performance measurements of physical activities. The sensor also shows good stability due to the simple structure. We have checked that the sensor could function normally after at least 4 months.

For our current prototype device, we used wires to connect the sensor with the measurement circuit, and the sensor response was recorded using a scope-coder. Therefore, future work related to this study will include the development of a measurement system with a miniaturized circuit that can be integrated onto the eyeglasses to enable wireless data transmittance. Furthermore, because the pulse wave and respiration rate are extracted from the sensor signal using simple low-pass and high-pass filters in our proposed system, the development of such miniaturized circuits for signal processing would not be a critical problem in the fabrication of these devices.
